# Development of Multiple UAV Collaborative Driving Systems for Improving Field Phenotyping

**DOI:** 10.3390/s22041423

**Published:** 2022-02-12

**Authors:** Hyeon-Seung Lee, Beom-Soo Shin, J. Alex Thomasson, Tianyi Wang, Zhao Zhang, Xiongzhe Han

**Affiliations:** 1 Department of Biosystems Engineering, College of Agriculture and Life Sciences, Kangwon National University, Chuncheon 24341, Korea; hslee91@kangwon.ac.kr (H.-S.L.); bshin@kangwon.ac.kr (B.-S.S.); 2 Interdisciplinary Program in Smart Agriculture, College of Agriculture and Life Sciences, Kangwon National University, Chuncheon 24341, Korea; 3 Department of Agricultural and Biological Engineering, Mississippi State University, Starkville, MS 39762, USA; athomasson@abe.msstate.edu; 4 College of Engineering, China Agricultural University, Beijing 100083, China; timothywangty@tamu.edu; 5 Key Laboratory of Smart Agriculture System Integration, Ministry of Education, China Agricultural University, Beijing 100083, China; zhaozhangcau@cau.edu.cn; 6 Key Laboratory of Agriculture Information Acquisition Technology, Ministry of Agriculture and Rural Affairs of China, China Agricultural University, Beijing 100083, China

**Keywords:** multiple UAVs, remote sensing, collaborative driving, field phenotyping, synchronized motion

## Abstract

Unmanned aerial vehicle-based remote sensing technology has recently been widely applied to crop monitoring due to the rapid development of unmanned aerial vehicles, and these technologies have considerable potential in smart agriculture applications. Field phenotyping using remote sensing is mostly performed using unmanned aerial vehicles equipped with RGB cameras or multispectral cameras. For accurate field phenotyping for precision agriculture, images taken from multiple perspectives need to be simultaneously collected, and phenotypic measurement errors may occur due to the movement of the drone and plants during flight. In this study, to minimize measurement error and improve the digital surface model, we proposed a collaborative driving system that allows multiple UAVs to simultaneously acquire images from different viewpoints. An integrated navigation system based on MAVSDK is configured for the attitude control and position control of unmanned aerial vehicles. Based on the leader–follower-based swarm driving algorithm and a long-range wireless network system, the follower drone cooperates with the leader drone to maintain a constant speed, direction, and image overlap ratio, and to maintain a rank to improve their phenotyping. A collision avoidance algorithm was developed because different UAVs can collide due to external disturbance (wind) when driving in groups while maintaining a rank. To verify and optimize the flight algorithm developed in this study in a virtual environment, a GAZEBO-based simulation environment was established. Based on the algorithm that has been verified and optimized in the previous simulation environment, some unmanned aerial vehicles were flown in the same flight path in a real field, and the simulation and the real field were compared. As a result of the comparative experiment, the simulated flight accuracy (RMSE) was 0.36 m and the actual field flight accuracy was 0.46 m, showing flight accuracy like that of a commercial program.

## 1. Introduction

Owing to the recent breakthrough in unmanned aerial vehicles (UAVs) or drones, their applications in the agricultural field, such as in crop monitoring, detection of crop diseases, digital surface modeling (DSM), sowing, spraying, irrigation, and mapping, have significantly reduced working hours and labor requirements, greatly improving the efficiency of agricultural work. In particular, they are widely used for remote sensing [1,2]. Currently, remote sensing in the agricultural field mainly involves remote exploration using a single UAV system [3,4,5], mapping [6,7,8,9], monitoring [10,11], and DSM [12,13]. The remote sensing process varies depending on the features (characteristics) of crops, the cultivation environment, and the exploration purpose; however, it mostly uses ortho-images obtained using red–green–blue (RGB) cameras and multi-spectral cameras. After performing image registration based on the ortho-images captured by the UAV along the image acquisition path, a post-processing process (which involves calculating the normalized difference vegetation index (NDVI), the carotenoid reflectance index (CRI), the normalized difference red edge index (NDRE), etc.) is used according to the purpose. The agricultural UAV market is growing rapidly, and many commercial UAVs have been released. Major UAV companies include DJI, Parrot, Precisionhawk, and AGEagle, and they are developing products for a variety of agricultural solutions [5]. Most of the UAVs used for image acquisition utilize a multi-rotor (quadcopter or hexacopter) as the main platform, and an appropriate UAV is chosen based on the total payload and sensors attached [14]. Most commercial UAVs are controlled using a ground-installed flight control system called a ground control station (GCS). It coordinates and controls all commands sent to the UAV, as well as all data received from the UAV, and controls all conditions before, during, and after the flight. Commercial GCSs include open-source-based MissionPlanner and QgroundControl [15]. The GCS supported by a UAV varies depending on the flight control (FC) firmware installed in the UAV, and the commercial GCS also changes according to the FC selected by the user. MissionPlanner, which supports only ArduPilot and operates only on Windows-based systems, is suitable for professional use. QgroundControl has the advantage that it supports both ArduPilot and PX4, operates not only on Windows but also on most other operating systems (Linux, iOS, Android, etc.), and can be easily operated, even by beginners.

In general, UAVs are widely used for farm work, such as crop monitoring, control, and sowing, which are required for agriculture. As UAVs use batteries as their main power source, a single UAV has limited operating time, taking considerable time to cover large areas. Multiple images taken from different directions are required for the precision maps used in precision agriculture, and field phenotyping requires high accuracy [16]. Thus, the use of multiple drones rather than a single drone is more efficient for acquiring images of a large area in a short period of time [17]. Nevertheless, the commercial programs currently controlling UAVs are often developed based on single-center flight control. Even when multiple UAVs can be controlled, these programs usually do not provide real-time synchronized control and independent flight control. Research is underway to solve this problem and enable a large number of UAVs to fly in groups [18,19]; moreover, research has been actively conducted for the cluster flight of a large number of UAVs in agriculture. Ju and Son used seven measurement indices (total time, setup time, flight time, battery consumption, inaccuracy of land, haptic control effort, and coverage ratio) to evaluate the performance of single and multiple UAVs during agricultural work, and proved that the multi-UAV system is more efficient than the single UAV system (total flight time improved by 18.1%, battery consumption reduced by 59.8%, coverage area increased by 200%, etc.) [20]. Barrientos et al. subdivided a large area of agricultural land using multiple UAVs and applied an efficient flight strategy, suggesting the application to real large agricultural lands through real field flight experiments [21]. A more complex control system is required for controlling multiple UAVs compared to a single UAV, and various constraints must be considered. The basic flight control for multiple UAVs is the same as for a single UAV, using a computer or remote controller with appropriate flight control software, such as GCS. The development of multiple path generation and multiple UAV collaborative driving algorithms is necessary to enable multiple UAVs to fly simultaneously. Several studies have been conducted on the efficient path planning and reliable flight control of multiple UAVs. Roberge et al. demonstrated that applying a genetic algorithm and a particle swarm optimization algorithm to a multi-core CPU allows UAV path planning within 10 s [22]. Gu et al. combined a wireless sensor network and multiple UAVs to enable efficient collaborative operation, and proposed a method for recognizing objects and locations using multiple UAVs [23]. Lee et al. performed multi-UAV control using the robot operating system (ROS) global positioning system (GPS) waypoint tracking package and a centralized task allocation network system. To overcome the battery limitations of a single UAV, this system was developed to enable multiple UAVs to take turns and complete continuous flight missions [24].

In general, in the case of UAVs applied to agriculture, scanning is carried out over a large area. Such remote sensing can be used to accelerate the arduous process of performing crop inventory and yield estimates for large areas. Area sizes typically used for remote sensing in agriculture, ranging from 5 to 10 hectares, are used for remote sensing and generating field phenotypes [25]. However, they have difficulties in providing high accuracy for key phenotypic characteristics, such as stem diameter, leaf angle, crop height, and leaf area index (LAI), as a result of the low spatial resolution of the data due to flight height. To solve this issue, phenotyping studies based on unmanned ground vehicles (UGVs) are in progress. Young et al. measured corn height and stem width using a stereo camera and a time-of-flight depth sensor mounted on a UGV. The field phenotyping showed a measurement error of 15% for the plant height and 13% for the plant stem width [26]. Madec et al. conducted a comparative experiment on the plant phenotypic characteristics observed by a UGV equipped with light detection and ranging (LiDAR) and a UAV equipped with RGB cameras. Although LiDAR showed higher phenotyping accuracy due to spatial resolution differences (LiDAR (3–5 mm), RGB (10 mm)), both LiDAR (UGV) and RGB (UAV) showed a high correlation with respect to plant height [27]. As UGVs can perform capturing under the canopy, important data, such as crop height and stem diameter, can be acquired throughout the development stage of the crop [28]. Manish et al. generated a mobile mapping system using a UGV. The developed UGV was equipped with LiDAR and RGB cameras, along with global navigation satellite system (GNSS)/inertial navigation system (INS) devices to align and project each coordinate system. A comparative experiment using a UAV and PhenoRover was also conducted to compare the accuracies. The accuracy of the point cloud generated in the UGV was ±5–8 cm, similar to that of the control group (UAV, PhenoRover), and the developed UGV showed a relatively low noise level during data collection, being able to capture individual plants [29].

Many studies have been conducted on measuring field phenotypes (crop height) using drone-based remote sensing. Christiansen et al. created a point cloud by combining the GNSS and an inertial measurement unit with LiDAR data. The created point cloud was mapped and analyzed using the functions of ROS and Point Cloud Library. Based on the analyzed crop height and volume, it was possible to estimate crop yield and nitrogen concentration [7]. Torres-Sánchez et al. collected images at flight altitudes of 50 m and 100 m to generate a DSM. As a result of image acquisition and accuracy analysis with different front and side overlaps for each altitude, it was possible to achieve 95% accuracy and 85% time saving by using 95% of front overlap and 60% of side overlap at a 100 m flight altitude [30]. Holman et al. collected images using a UAV equipped with an RGB camera and reconstructed the three-dimensional (3D) terrain through structure from motion (SfM). The height accuracy of the crop was $R^{2}$ ≥ 0.92, and the root means square error (RMSE) ≤ 0.07 m, showing high accuracy. When compared to terrestrial LiDAR (terrestrial LiDAR accuracy: $R^{2}$ = 0.97, RMSE = 0.027 m) for comparative experiments, although the SfM method using RGB did not show as high a level of accuracy as that of LiDAR, it seemed more suitable in terms of time and cost effectiveness for the wide areas of agricultural sites because LiDAR is expensive and requires a long scan time [31]. However, measurement errors may occur as the SfM method generates high-resolution 3D topography or field phenotyping from high-resolution images. Inaccurate GPS positioning accuracy [32] and plant movement due to wind or the rotor movement of UAVs [33] are considered to be the main causes of errors. To reduce measurement error and minimize the effect of the disturbance, images should be simultaneously acquired from multiple perspectives. However, the commonly used commercial GCS programs simply assign the pre-designed missions to single and multiple UAVs for images collected from a single perspective. As the UAVs are operated independently to complete the designated mission areas, it is impossible to acquire images simultaneously from multiple perspectives by collaborating with the multiple UAVs to obtain high-precision field phenotypes.

Many studies are underway to reconstruct the 3D phenotype models of crops using images taken from multiple perspectives [34,35,36,37,38], as they result in higher accuracy than by using images taken from a single angle, allowing the measurement of major organs of the crop (leaf area, plant height, crown area, calyx size, etc.) required for crop monitoring. Zhu et al. observed the morphological changes (plant height, plant length, plant width, crown height, crown area, etc.) of soybean plants based on low-cost 3D reconstruction technology, and the correlation coefficient obtained was significantly higher than 0.98, indicating high accuracy [34]. He et al. used strawberry images taken from multiple angles (360°), and rapidly obtained a quantitative phenotypic analysis of external strawberry traits, such as height, length, width, and the calyx size of strawberries [36]. Zermas et al. created a 3D point cloud using high-resolution RGB images collected from multiple angles (circular orbits) using a UAV and a portable camera, and measured the LAI, individual, and average plant height, leaf angle with respect to the stem, and leaf length. To overcome the limitations of two-dimensional (2D) images, a 3D point cloud was reconstructed by capturing images from multiple perspectives, which allowed the measurement of the main parts of the plant in detail with excellent accuracy (LAI accuracy of 92.48%, plant height accuracy of 89.2%, and leaf length accuracy of 74.8%) [38]. High-resolution images taken from multiple perspectives are required to improve the 3D model (phenotype) measurement accuracy and measure the major organs of crops.

This study aimed at developing a system for the collaborative driving of multiple UAVs to improve the accuracy of field phenotyping using UAV-based agricultural remote sensing, thus optimizing the cost, efficiency, and productivity, while reducing the impact on the external environment. The specific purposes of this study were: (1) to design, build, and test multiple UAVs; (2) to develop an algorithm to enable the collaborative driving of multiple UAVs; and (3) to verify the multiple UAV collaborative driving algorithm thus developed and optimize the variables, with a comparative verification experiment conducted by comparing it with the commercial GCS system in the simulation environment and the real world.

## 2. Materials and Methods

In this study, two unmanned aerial vehicles were produced, and a cooperative driving algorithm and collision avoidance algorithm were developed for stable swarm flight to improve phenotyping. After optimizing and verifying a number of unmanned aerial vehicles to which the developed algorithm is applied in the simulation environment, the same algorithm was applied in the real field to compare the simulation and real-world performance. In addition, a performance comparison with commercial programs was conducted. Flight accuracy and total flight time were used as indicators for algorithm evaluation and performance comparison.

### 2.1. System Architecture and Principles

#### 2.1.1. UAV

**Figure 1:**
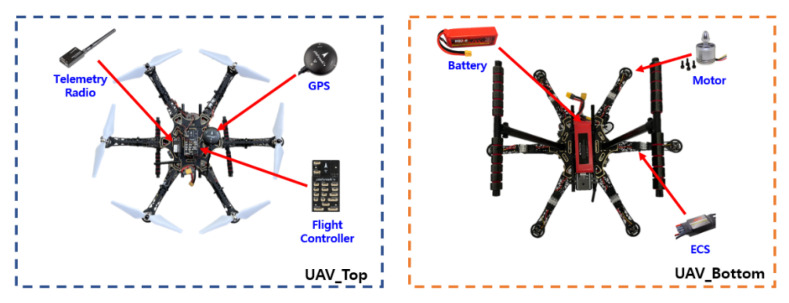
Main components of the collaborative unmanned aerial vehicle.

Figure 1 shows the overall structure of the system. The UAV uses a hexacopter as a base and is equipped with a flight control (Pixhawk 4, Holybro, Hongkong, China) to control aircraft attitude and flight, a GPS (Neo-M8N GPS, Ublox, Thalwil, Switzerland) to receive location information, an ECS (MR-X3, PolyTronics, Taiwan, China) to control rotor speed and direction, and three motors (S2312-920KV, PolyTronics, Taiwan, China) each for clockwise (CW) and counterclockwise (CCW). The GCS (PC) and each UAV are connected wirelessly by radio (Telemetry Radio V3 433Mhz, Holybro, Hongkong, China), and different IDs are assigned to prevent crosstalk. As for the battery (HBZ-B 4200 mAh 4S 35C, HBZ-B, Shijiazhuang, China), a Lipo 4S 4200 mAh is attached considering the payload and flight time. With all the equipment mounted, the maximum flight time is approximately 12 min.

#### 2.1.2. MAVLink Router

The UAV’s status information, location/attitude information, and control commands are transmitted and received between each UAV and the GCS (PC) through wireless communication (telemetry radio). Because telemetry radio supports only universal asynchronous receiver–transmitters (UART; serial communication), parallel communication is difficult. To use the UAV monitoring and control programs simultaneously in this study, the UART communication was changed to user data protocol (UDP) communication using the micro air vehicle link (MAVLink) router. MAVLink is a protocol for communicating with small, unmanned devices and different internal components within itself, and provides reliable data exchange [39]. The transmission rate of UART was 57,600 bps, and a total of four IP addresses (192.168.10.1, 192.168.10.2, 192.168.11.1, 192.168.11.2) were allocated and used.

#### 2.1.3. Ground Control Station (GCS)

**Figure 2:**
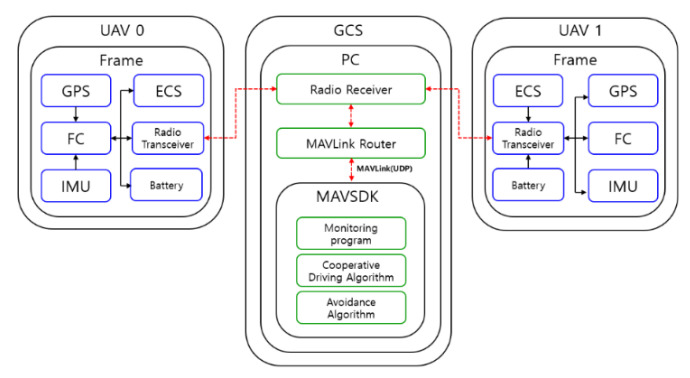
Structure of the unmanned aerial vehicle’s control system.

The GCS developed in this study receives the status and location information of the UAV through wireless communication (radio receiver), and converts the transmitted and received information into the UDP format through the MAVLink router. Each UAV is given a unique IP address and a port number, and each UAV’s status and location information are transmitted through the UDP based on the MAVLink protocol. The GCS developed based on MAVSDK uses the received location and status information of the UAV received via the UDP to implement and operate real-time UAV monitoring, the collaborative driving of multiple UAVs, and the avoidance algorithm. GCS development and operation were constructed in the Ubuntu 18.04.6 environment, and it was confirmed that GCS and UAV were wirelessly connected to each other for monitoring and control. Figure 2 shows the overall system structure, including the GCS. The GCS program developed in this study was written in Python (3.6.9), based on the MAVLink protocol.

### 2.2. Multiple Path Generation Algorithms

**Figure 3:**
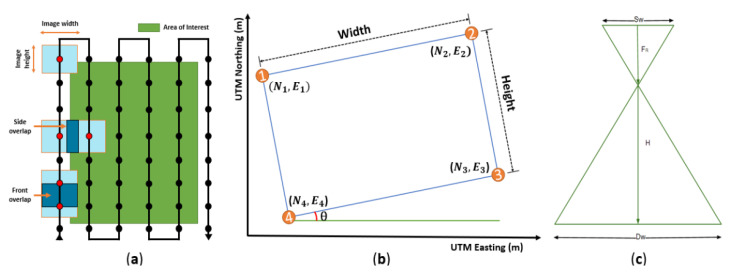
Variables to consider when generating a path: (**a**) an example of the overall path; (**b**) vertex of the area to be captured; (**c**) correlation of camera capture.

**Figure 4:**
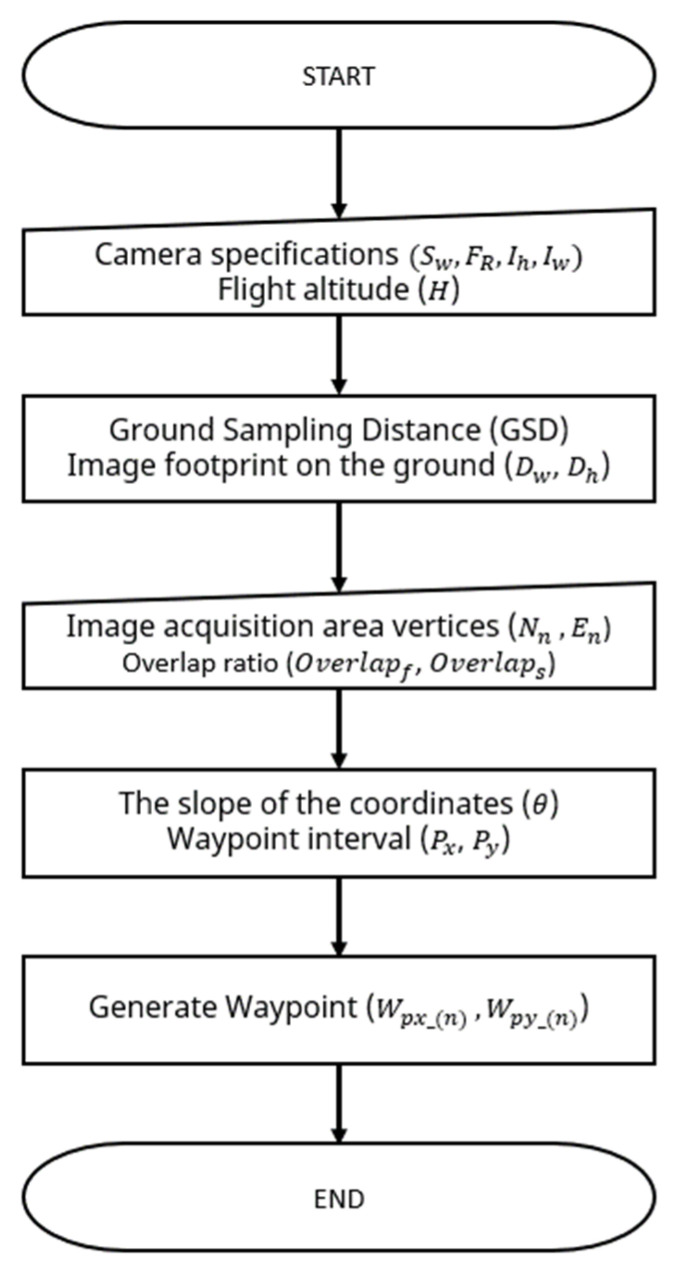
Flowchart of the path generation algorithm for the collaborative driving system.

In the operation of multiple UAVs, a unique flight path must be generated for each UAV. Many variables (flight altitude, camera specifications, field coordinates, and overlap ratio) must be considered to generate flight paths that are optimized for the image acquisition area and multiple UAVs. To create a waypoint, the image size and overlap ratio among both forward and side directions were considered, as shown in Figure 3a, and the field area was calculated based on the UTM coordinates, as shown in Figure 3b. Finally, the geometrical correlation between camera specifications and flight altitude was also considered, as shown in Figure 3c. Figure 4 shows the flowchart of the path generation. The camera’s specifications ($S_{w},\;F_{R},\;I_{h},\;I_{w})$ and the flight height (H) are input to determine the ground sample distance (GSD) and the actual image capture size on the ground ($D_{w}$, $D_{h}$) (Equations (1)–(3)). The overlap ratio and GSD are used to calculate the spacing between waypoints ($P_{x}$, $P_{y}$) (Equations (4) and (5)). When the coordinates ($N_{n},\;E_{n})$ of the actual image acquisition location are entered, the slope (θ) of the coordinates is calculated, and the waypoints ($W_{px\_\left(n\right)},\;W_{py\_\left(n\right)}$) are generated (Equations (6)–(7)). When the entire flight path of one UAV is generated, it is copied laterally based on the flight spacing to generate the flight paths of multiple UAVs. The universal transverse Mercator (UTM) coordinate system is used for all flight paths. Table 1 shows the input variables used in the multi-travel path generation algorithm developed in this study.

(1) $$GSD=\frac{S_{w}\times H\times 100}{F_{R}\times I_{w}}$$

(2) $$D_{w}=\frac{GSD\times I_{w}}{100}$$

(3) $$D_{h}=\frac{GSD\times I_{g}}{100}$$

(4) $$P_{x}=D_{w}\times \left(1-Overlap_{f}\right)$$

(5) $$P_{y}=D_{h}\times \left(1-Overlap_{s}\right)$$

(6) $$\theta =\tan^{-1}\;\left(\frac{N_{2}-N_{1}}{E_{2}-E_{1}}\right)$$

(7) $$W_{px\_\left(n+1\right)}=W_{px\_n}+\left[(P_{x}\times \cos\theta )-\left(P_{y}\times \sin\theta \right)\right]$$

where $W_{px_{n}},\;W_{py_{n}}$ is the flight path point (X,Y) of UAV (m), $Overlap_{f}$ and $Overlap_{s}$ are front and side image overlaps (%), respectively, $P_{x},P_{y}$ are waypoint point X,Y intervals (m), $S_{w}$ is the sensor width of the camera (mm), $F_{R}$ is the focal length of the camera (mm), H is the flight height (mm), $I_{h}$ and $I_{w}$ are the image height and width (pixels), respectively, GSD is the ground sampling distance (cm/pixel), $D_{w}$ and $D_{h}$ are the width and height of a single image footprint on the ground (m), respectively, θ is the slope of the coordinates (°), and $N_{n},\;\;E_{n}$ are image acquisition area vertex coordinates (m).

### 2.3. Multiple UAV Collaborative Driving Algorithm

**Figure 5:**
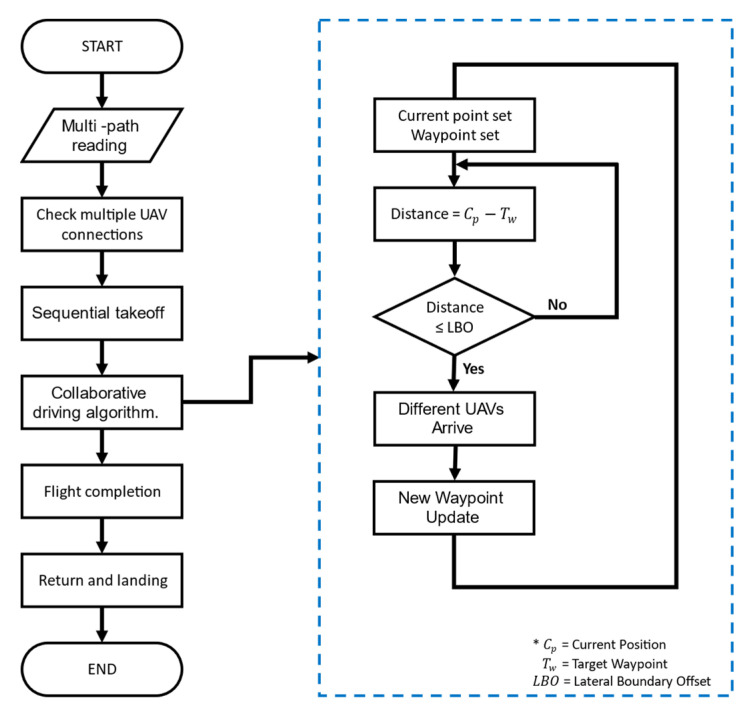
Collaborative driving algorithm of multiple unmanned aerial vehicles.

**Figure 6:**
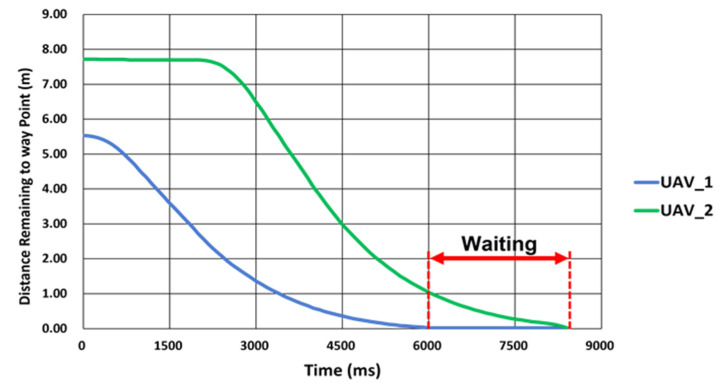
The altitude of each drone when waiting for different UAVs.

Multiple UAVs fly by following individually entered flight paths. The developed collaborative driving algorithm facilitates the following mechanism: the location information of each UAV is synchronized with the GCS; therefore, if external environmental factors (wind) or flight latencies of other UAVs occur, the UAVs can overcome them and fly according to the set paths while maintaining a certain distance and altitude. Figure 5 shows the overall flowchart for the flight. When the program starts, the path generated for each UAV is read. If the flight path reading is completed normally, it is checked as to whether the communication connection with each UAV has been established normally. If the wireless connections have been established normally, each UAV takes off sequentially. After takeoff, each UAV moves to the first waypoint sequentially. If UAV 1 satisfies Condition (1) and UAV 2 in the same row satisfies Condition (1) at the same time, new paths are updated for them simultaneously. Multiple UAVs update paths consecutively, and when they fly to the last waypoint, the flight ends, and they return automatically to the home location and land. Through the collaborative driving algorithm, the UAVs can simultaneously acquire the images required for field phenotyping by waiting for other UAVs and updating waypoints simultaneously, as shown in Figure 6.

**Condition** **1.** *Current location of UAV–target waypoint location ≤ LBO (lateral boundary offset).*

#### 2.3.1. Driving Waypoint Update

**Figure 7:**
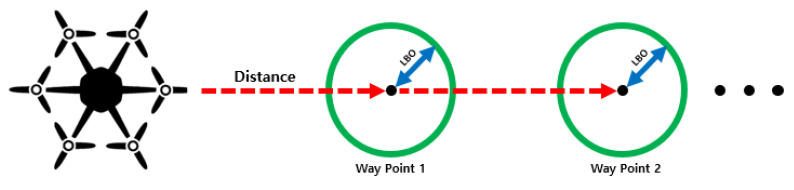
An example of driving waypoint update.

The path needs to be updated for the UAV to fly along the path to the last waypoint while updating the waypoints. Waypoint updates are included in the algorithm for multiple UAVs. As shown in Figure 7, the UAV approaches a waypoint as the target, and if it reaches the lateral boundary offset (LBO), it updates a new waypoint and continues following the path. The GCS calculates the relative distance between the waypoint and the UAV in real time and updates the path.

#### 2.3.2. Collision Avoidance

**Figure 8:**
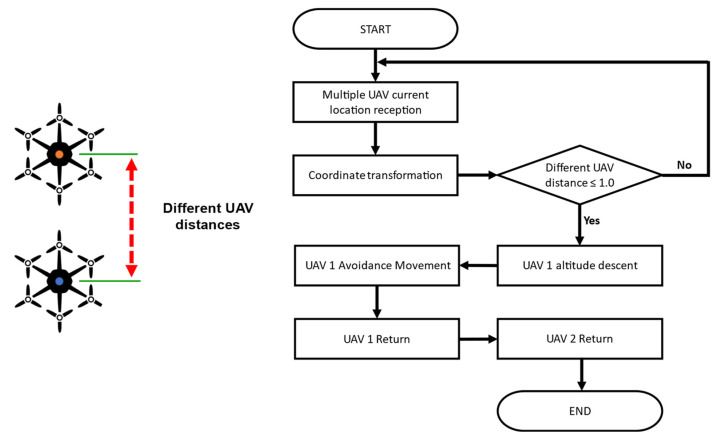
Flowchart of the collision avoidance algorithm in the collaborative driving of multiple UAVs.

The collision avoidance function of a general commercial unmanned aerial vehicle is designed mainly for obstacles. However, the risk of collision is very high because multiple UAVs fly in close proximity as a swarm along the crop row. Usually, the possibility of collision is small, but considering the possibility of two UAVs colliding due to external disturbance (wind) or GPS error, an algorithm to eliminate the risk was developed and applied in the collaborative driving of multiple UAVs: it avoids collision automatically when two UAVs are close enough to collide with each other. Figure 8 shows the flowchart of the collision avoidance algorithm. If the distance between two UAVs is closer than that of the flight plan, a collision warning is sent to the user, and then the altitude of UAV 1 is lowered by 5 m to that of UAV 2. Once UAV 1 descends the altitude, it moves to the left by 2 m and to the rear by 2 m from the current position of UAV 2. After moving, UAV 1 lands first, followed by UAV 2.

### 2.4. Flight Simulation

#### 2.4.1. Flight Simulation Configuration

**Figure 9:**
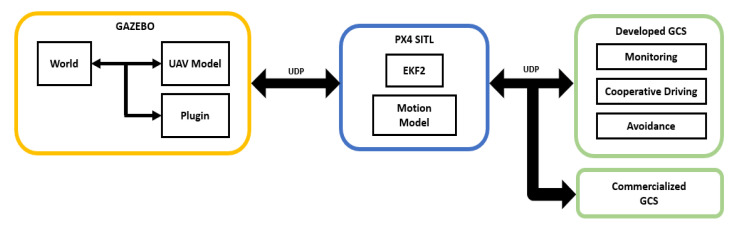
Configuration of the virtual simulation environment.

The developed algorithms were verified, and a virtual environment was constructed that could conduct a number of unmanned aerial vehicle simulation flight experiments under various conditions. GAZEBO 9, an open-source robot simulator, was used for the simulation environment. Figure 9 shows the overall structure of the simulation. The GAZEBO environment includes a virtual map and a UAV model, and the plug-ins required for the real operation of the UAV are additionally installed. The simulator (GAZEBO) and the software-in-the-loop (SITL) are interlinked and communicate using the MAVLink. The most commonly used UDP is utilized for MAVLink communication. The simulated location information and real-world location information can be linked with each other, and MAVLink-based commands can be used to control the actual movement and flight control of the UAV using PX4 SITL. At the same time, the developed GCS can be connected to enable monitoring and to apply the developed algorithms. Furthermore, multiple commercial GCSs can also be connected. All status information is saved in a flight log for each UAV. The flight log is used later in the optimization task of the algorithms.

#### 2.4.2. Check and Optimize the Developed Algorithm

The algorithm (multiple path generation, collaborative driving of multiple UAVs, and collision avoidance) developed in this study was checked and optimized. As the multiple-path generation algorithm and the collaborative driving algorithm of multiple UAVs are operated in conjunction, they were checked and optimized together. Optimization was performed for the size of the LBO and the maximum flight speed, which have the greatest influences on the driving algorithm. For the collision avoidance algorithm, virtual collision paths were generated to check whether the UAVs operate normally according to the algorithm.

#### 2.4.3. LBO Optimization

The size of the LBO is the variable that has the greatest influence on the waypoint updates. If the LBO is large, the waypoint is updated quickly, but the flight location error would be large, as the path is updated far away from the waypoint. In contrast, if the size of the LBO is small, the waypoint update takes more time, but the location error is small because the path is updated close to the waypoint. Because it is difficult to fly under the same conditions every time in the field, the LBO size was set to 0.2 m intervals in three steps (0.2, 0.4, 0.6) in the flight simulation environment, and the flight log was analyzed after completing the flight. Then, the size of the LBO was selected considering the total flight time and position error.

#### 2.4.4. Flight Speed Optimization

The maximum flight speed is a variable that affects the path updates, such as the LBO, as well as the total flight time. An appropriate maximum flight speed needs to be set based on the distance interval between the waypoints. Basically, the UAV decelerates the flight speed as it approaches the waypoint. However, if the maximum flight speed is too high, the deceleration time will be high and the UAV will pass by the waypoint, resulting in a loss of flight time. In contrast, if the maximum flight speed is too low, the total flight time will increase, resulting in reduced flight efficiency. In the simulation environment, the maximum flight speed was set at an interval of 2.0 m/s in three steps (1.0, 3.0, 5.0), and the flight log was analyzed after the flight was completed. After that, the optimum maximum flight speed was selected in consideration of flight position error and total flight time.

#### 2.4.5. Avoidance Algorithm Verification

Conducting a collision experiment with the initially developed collision avoidance algorithm in a real-world environment would involve a very high level of risk. Hence, the algorithm was firstly tested in the developed simulation environment. Multiple UAVs were configured to fly normally along the paths and then collide with other UAVs by arbitrarily modifying the paths. Here, the flight logs of multiple UAVs recorded in the simulation were analyzed to check whether they avoided collisions normally and returned and landed at home according to the collision avoidance algorithm.

### 2.5. Field Test

#### 2.5.1. Long-Distance Wireless Communication Latency

**Figure 10:**
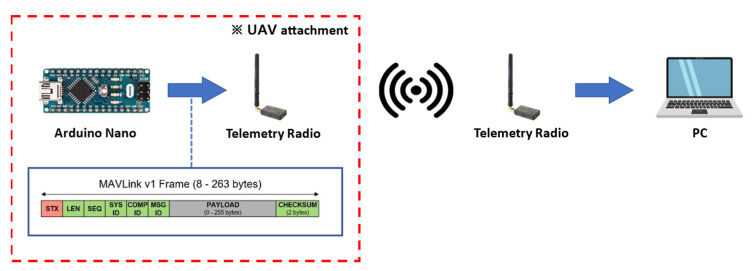
A configuration diagram of the long-distance wireless communication latency experiment.

Communication latency is unavoidable in long-distance wireless communication. Therefore, the experiments were conducted on long-range wireless communication between GCS and UAV in real field environment. In this study, a Telemetry Radio V3 433Mhz (the same communication device used in this study) and an additional microcontroller were attached to the UAV to measure the loss rate and latency in radio communication. Figure 10 shows the structure of the communication latency measurement system. The microcontroller automatically generates an arbitrary virtual message based on the MAVLink v1 frame. The virtual message is written as a random string with a size of 130 bytes. The generated message is transmitted to the telemetry radio through UART, and it is then sent wirelessly to the receiving telemetry radio connected to the PC. The virtual message is sent in a constant transmission cycle (100 ms) using the timer interrupt of the microcontroller, and the receiver (PC) receives it while recording the time. The latency time is calculated by using Equation (8). The communication delay can be calculated by subtracting the transmission cycle from the difference between the time of receiving the previous data and the time of receiving the next data. The measurement experiment was conducted in an open field (a lot in front of the College of Agriculture and Life Sciences, Kangwon National University) with the measurement device-attached UAV hovering at an altitude of 30 m. The communication latency time was calculated as a 10 s average of the latency time.

(8) Latency (ms)=(After data reception time − Previous data reception time)−100 ms

#### 2.5.2. Multiple UAV Collaborative Driving Algorithm Verification

**Figure 11:**
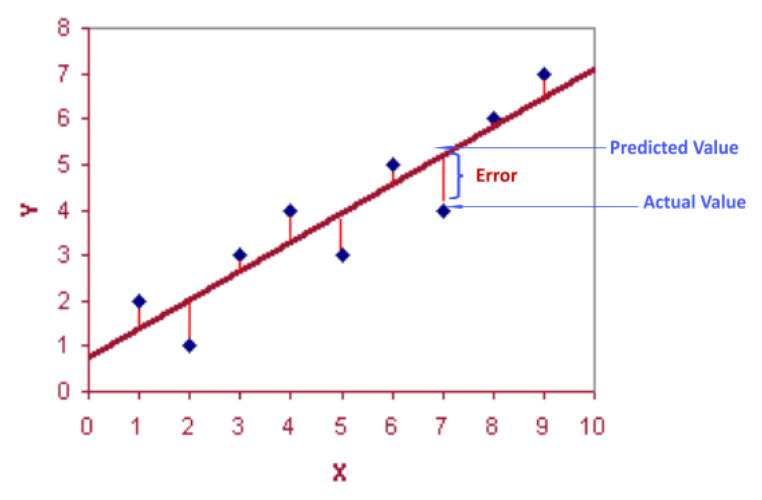
Flight accuracy measurement.

The flight experiment was conducted by applying the multiple-path generation algorithm and the collaborative driving algorithm of multiple UAVs—which were checked and optimized in advance through simulations—to the real field. The experiment was conducted on the sports field (open field) of the Kangwon National University Medical School, and Table 2 shows the experimental conditions and flight parameters. The saved flight log was analyzed after the completion of the entire flight. The flight trajectory and flight altitude information were extracted from the flight log and compared to the generated multiple flight paths (Figure 11). For flight (location) accuracy analysis, the straight flight sections, excluding the rotating flight sections, were analyzed with RMSE (Equation (9)).

(9) $$\mathrm{RMSE}=\sqrt{\frac{1}{n}\overset{n}{\underset{i=1}{\sum }}{\left(f_{i}-o_{i}\right)}^{2}}$$

where f is the predicted value (m), o is the actual value (m), and n is the total sample size.

#### 2.5.3. Collision Avoidance Algorithm Verification

**Figure 12:**
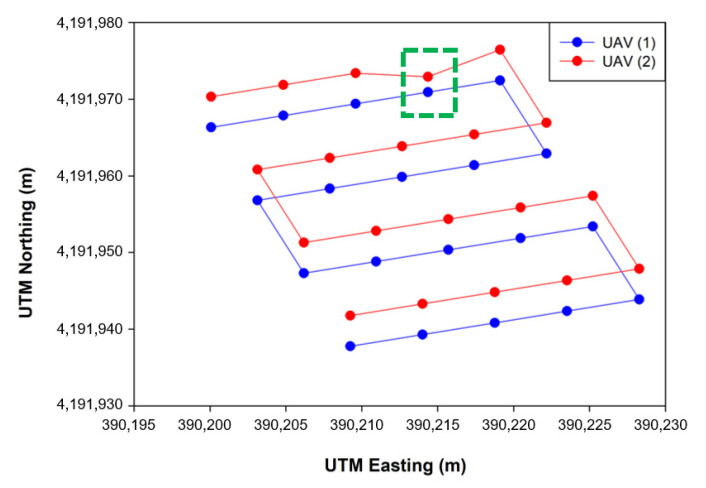
Flight path to verify collision avoidance algorithm.

The flight experiment was conducted by applying the collision avoidance algorithm, which was verified in advance via simulation, to the real field. To perform collision verification arbitrarily, the collision path was generated as shown in Figure 12. The fourth waypoint was modified arbitrarily so that two UAVs would be close to each other within 2 m. The place of the experiment was the sports field (open field) of the Kangwon National University Medical School, and Table 3 shows the experimental conditions and flight parameters. After the collision avoidance flight was completed, the saved flight logs were analyzed. Flight trajectories and flight altitude information were extracted from flight records to determine whether the avoidance proceeded normally according to the collision avoidance algorithm.

#### 2.5.4. Comparison with Existing Commercial Programs (GCS)

Currently, an improved QgroundControl is being developed that enables more efficient mission planning than the existing commercial program (QgroundControl) and allows operators to build complex missions [40]. In general, to perform autonomous flight and image acquisition using the commonly used commercial GCS programs (QgroundControl and MissionPlanner), flight parameters, such as field coordinates, camera specifications, flight altitude, flight speed, and image overlap ratio, should be manually input to the GCS programs in consideration of the GSD, flight time, etc. The flight parameters are often changed according to the user’s needs for the image acquisition, rather than for the well-parameterized values in the GCS program during the UAV flights. However, in this study, programs and algorithms were developed to implement essential functions, such as stopping during flights and waiting for different UAVs, to improve phenotyping or collision avoidance. Additionally, since many users generally use the existing programs the most, only the program developed in this study and the commercial program were compared with the same flight parameters. To compare the driving performance between the developed GCS and an existing commercial program, a flight comparison experiment was conducted by using the same flight path in the developed GCS and commercial GCS (QgroundControl). All flight parameters (flight altitude, maximum flight speed, LBO size, etc.) were set the same, and because the commercial GCS lacked the collaborative driving function, only a single UAV was used in the comparative experiment. The commercial GCS performed unmanned flight in mission mode by inputting the flight path in the UAV in advance. The developed GCS, on the other hand, performed unmanned flight by applying the collaborative driving algorithm. After the flight was completed, the flight log was analyzed, and each flight trajectory (accuracy) and flight time were compared.

#### 2.5.5. Comparison between Real and Simulated Environments

A comparative experiment was conducted for the cooperative operation of multiple UAVs in a flight simulation environment and in a real field. The simulation was configured to be as similar to the real field experiment as possible by adding an operation latency corresponding to the actual measured wireless communication latency time. All flight parameters, including the optimal flight parameters (LBO, maximum flight speed) found earlier, and multiple flight paths were set using the same conditions (Table 4). After completing all flights, the flight log and actual site recorded in the simulation were analyzed to investigate the flight time and flight trajectory (accuracy).

## 3. Results and Discussion

### 3.1. Results of Multiple Path Generation Algorithms

**Figure 13:**
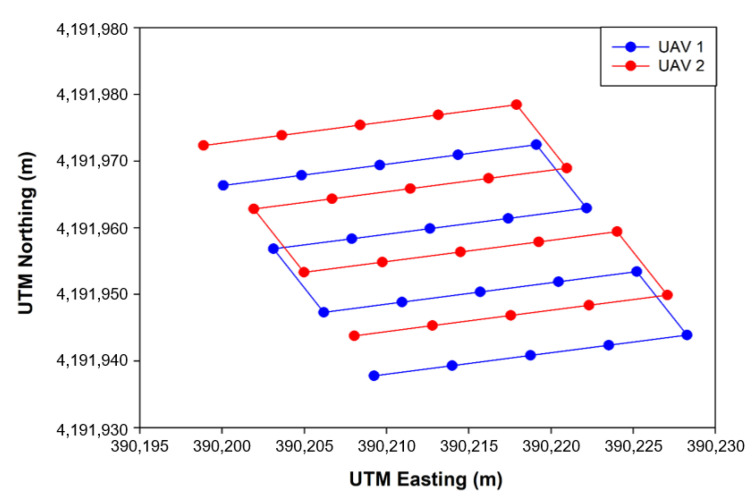
Multiple flight path generation obtained from the multiple-path generation algorithm.

Multiple paths were generated with the multiple-path generation algorithm developed in this study. Prior to flying, all parameters required for the flight path generation were input to generate a path for each UAV. Figure 13 shows the flight paths used in the algorithm verification and field experiment conducted in this study. Multiple paths were generated with 20 waypoints, a waypoint interval of 5 m, a total flight distance of 110 m, and a coordinate slope of 17.8° for each UAV. All waypoints were generated above the sports field of the Kangwon National University Medical School, and the UTM coordinates of Zone 52S were used.

### 3.2. Results of Flight Simulation

#### 3.2.1. Multiple UAV Collaborative Driving Algorithm Optimization

**Figure 14:**
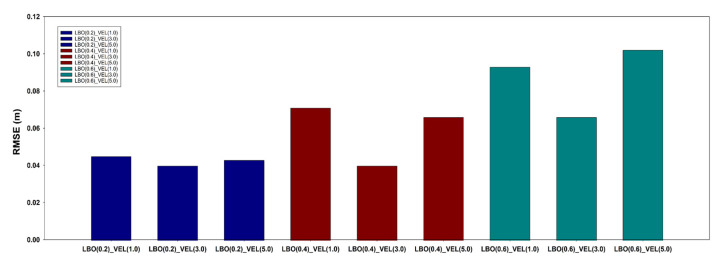
Flight accuracy by flight variable.

**Figure 15:**
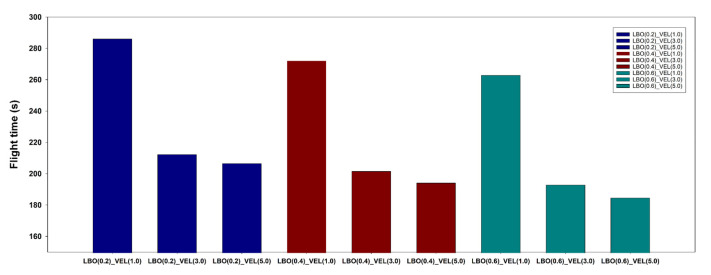
Total flight time by flight variable.

Variable optimization for the multiple UAV collaborative driving algorithm was performed in the simulation environment developed for this experiment. In the variable optimization, nine variable combinations were used, with three levels (1, 3, and 5) of speed (m/s) and three levels (0.2, 0.4, and 0.6) of LBO (m). As shown in Figure 14, the flight accuracy increased with a decrease in the LBO size. As shown in Figure 15, the flight time decreased with an increase in the maximum flight speed. First, the maximum flight speed had the greatest effect on the total flight time, whereas the size of the LBO considerably affected the flight accuracy. The variable combinations with the highest flight accuracy were the LBO size of 0.2 m with a maximum flight speed of 1 m/s and the LBO size of 4.0 m with a maximum flight speed of 3.0 m/s. The combination of variables with the shortest total flight time (185 s) was the LBO size of 0.6 m with a maximum flight speed of 5 m/s, although the flight accuracy was the lowest at 0.102 m. As the goal of the UAV operation in this study was to collect images at an accurate location, priority was given to achieving high flight accuracy. Considering the total flight time as a second priority, the combination of the LBO size of 0.4 m and a maximum flight speed of 3 m/s was selected as the optimal combination of variables for achieving the highest flight accuracy with the shortest total flight time.

#### 3.2.2. Results of Collision Avoidance Algorithm Verification

**Figure 16:**
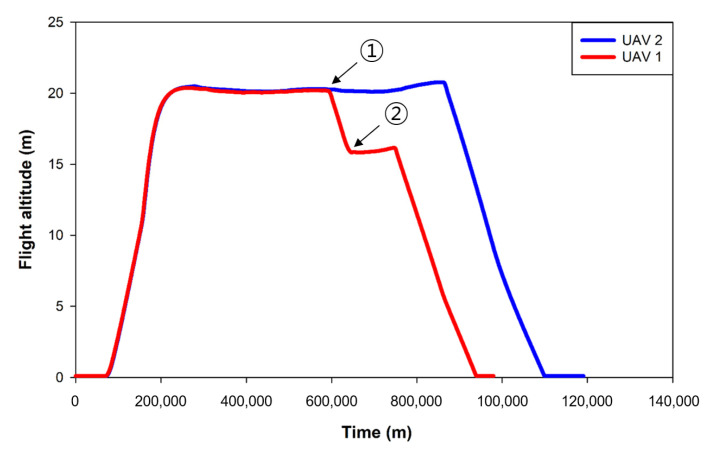
Flight altitude for each UAV during collision avoidance.

**Figure 17:**
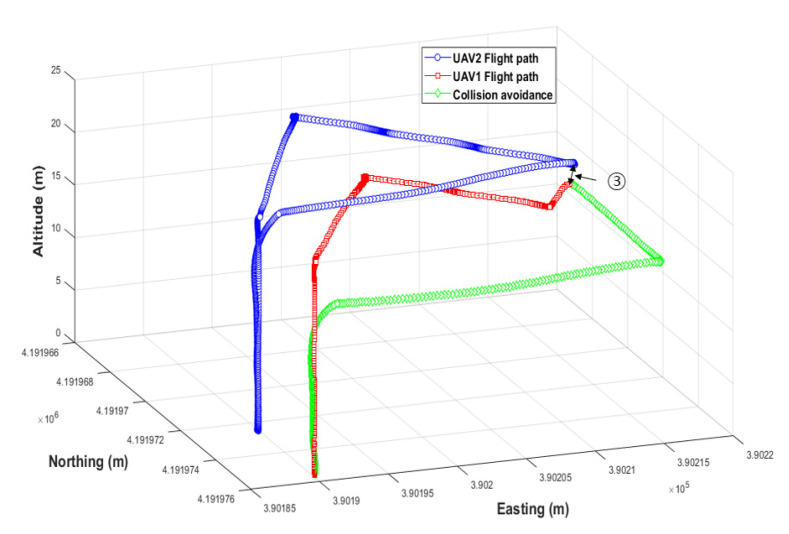
Simulation of flight trajectory with the collision avoidance algorithm.

In the simulation environment, a large number of UAVs flew along a modified path, and different UAVs were induced to randomly collide. As shown in Figure 16-①, the collision avoidance algorithm operated normally before the collision, and the collision was avoided. In the collision avoidance process, UAV 1 lowered the altitude as shown in Figure 16-② and, as shown in the avoidance trajectory in Figure 17-③, UAV 2 moved to the left and rear. Both UAVs safely returned to their home positions. In this experiment, a collision situation was induced by modification, but UAVs may collide while flying close to one another due to disturbance (wind) or unknown reasons during the operation of multiple UAVs. Therefore, collision accidents could be prevented by applying the developed collision avoidance algorithm. As a result of the collision test in the simulation environment, the maximum proximity distance between UAVs was found to be 1.01 m, although the collision avoidance detection distance was set to 2.0 m. The collision avoidance latency may have been due to the inaccurate location of the UAV detected by the GPS noise module installed in the simulation. Thus, improving the GPS noise and selecting an appropriate collision distance according to GPS accuracy is necessary before applying it in a real field.

### 3.3. Results of Field Test

#### 3.3.1. Wireless Communication Latency

**Figure 18:**
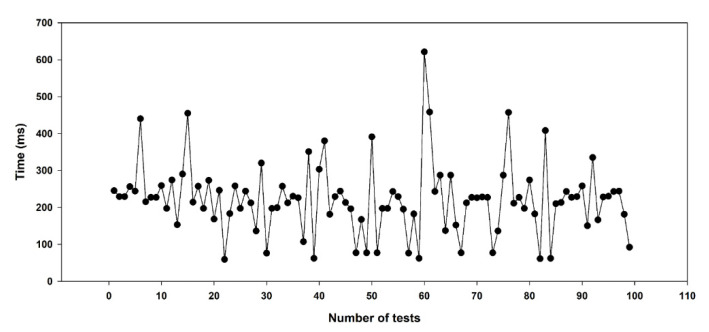
Time latency results of long-distance wireless communication test.

Figure 18 shows the results of the latency experiment for the wireless communication between the UAV and the GCS. The average latency was 334 ms, and there was no loss of wireless communication. When wireless communication between the UAV and the GCS was performed using MAVLink in a similar wireless communication experiment, the wireless communication latency was 420 ms, with an average packet loss of 0.63% for a communication distance of 20 m [31]. The average latency of 334 ms and the loss rate of 0% shown in our communication latency experiment were sufficient for the remote control of the UAV using MAVLink.

#### 3.3.2. Comparison between Simulation and Actual Field Tests

**Figure 19:**
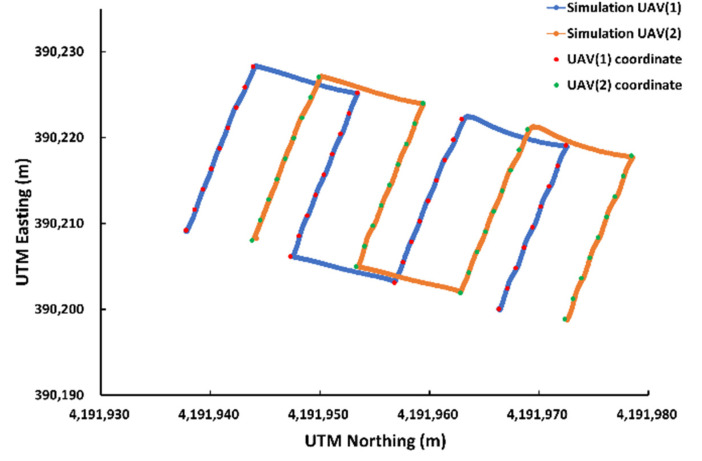
Flight trajectories obtained from the two UAVs in the simulation environment.

**Figure 20:**
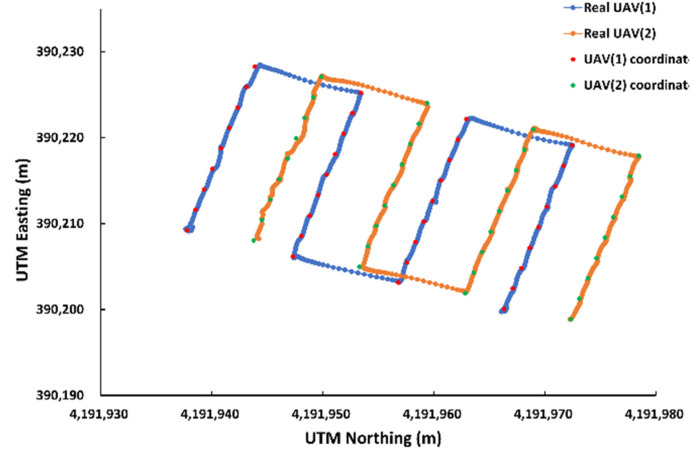
Flight trajectories obtained from the two UAVs in the actual field.

As a result of flight comparison experiments in the simulated environment and real field with the optimized flight variables (an LBO size of 0.4 m and a maximum flight speed of 3 m/s), the flight trajectory was as shown in Figure 19 and Figure 20. As for the simulation, the total flight time was 164 s and the flight accuracy was 0.06 m, whereas for the actual flight, the total flight time was 185 s and the flight accuracy was 0.08 m (Table 5). The total flight time used here was calculated as the total flight time of UAV 1 from the first waypoint to the last waypoint, and the flight accuracy was extracted from a total of four straight-section flights. When flying on the same path, the flight time was longer in real flight with a lower flight accuracy than in the simulation environment. As there was no external disturbance in the flight in the simulation environment, there was less shaking during the flight than when flying in the actual field, leading to smooth waypoint updates as well as a shorter total flight time. As the developed simulation reproduced the flight paths and tendencies similar to flight experiments conducted in the actual field, it could be used as a tool to verify the simulation flight and algorithm prior to the actual flight.

#### 3.3.3. Collision Avoidance in the Actual Field

**Figure 21:**
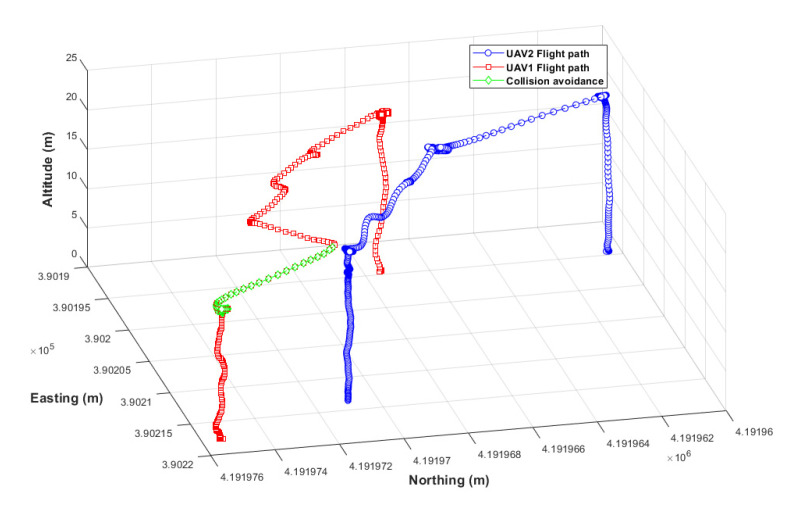
Actual flight trajectory with the collision avoidance algorithm.

As a result of the collision avoidance verification experiment in the actual field, using the collision avoidance algorithm verified in the simulation environment and the arbitrarily modified collision path, collision avoidance was normally performed, as shown in Figure 21. The maximum proximity distance of the UAVs was 0.33 m, and the collision avoidance response was delayed by 0.68 m compared to the simulation. As long-distance communication latency occurred along with GPS position error in the actual field, collision detection responded later than in the simulation. For a faster collision avoidance response, a long-distance communication module with low latency and a real-time kinematic global positioning system (RTK-GPS) with higher accuracy than general GPS seems necessary.

#### 3.3.4. Comparison of Existing Commercial and Developed Program Performance

**Figure 22:**
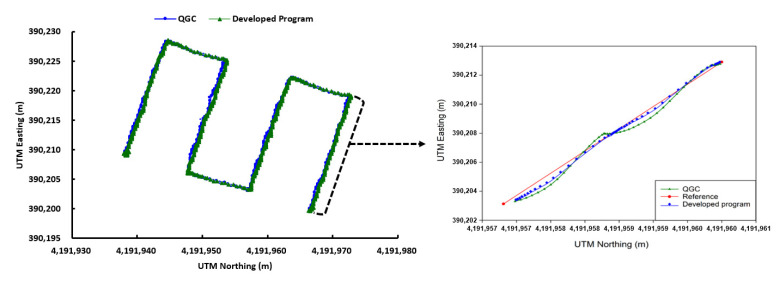
Comparison of commercial and developed program flight trajectories.

Figure 22 shows the actual flight trajectories of the UAV to which the collaborative driving algorithm developed in this study was applied and the UAV using the commercial program. Although the flight trajectories appeared similar to each other, the flight accuracy was measured to be high at 0.08 m for the UAV with the collaborative driving algorithm applied. The trajectory of the UAV operated by the commercial program (QgroundControl) was S-shaped, fluctuating up to 0.15 m or more from the original path, whereas the UAV controlled by the developed program stably followed the waypoints (fluctuating up to 0.09 m from the reference path). The total flight time was shorter with the commercial program, at 127 s, than with the developed program (Table 6). The commercial program did not support collaborative driving, and the flight time was shorter than when using collaborative driving as the UAV did not have to wait for other UAVs, and could simply move on after updating the waypoints on its own. The autonomous flight of a number of UAVs to which a multiple UAV collaborative driving algorithm was applied involved a loss in flight time compared to the existing commercial programs. However, it seems more suitable for capturing images to improve phenotyping, as two or more UAVs can operate collaboratively and provide higher accuracy.

## 4. Discussion

This paper developed a system for the cooperative driving of multiple UAVs to improve the accuracy of on-site phenotyping and to reduce external environmental influences when using UAV-based agricultural remote sensing. As depicted in Figure 20 and Table 5, the collaborative driving algorithm was verified in the simulation environment and in the actual field. A simulation flight accuracy of 0.06 m, a field flight accuracy of 0.08 m, a simulated flight time of 164 s, and a field flight time of 185 s revealed a similarity between the simulation results and the field results, demonstrating the reliability of the flight simulation. Through collaborative flight, it was possible to fly while waiting for different delayed UAVs, and a collision avoidance algorithm that could occur when flying in clusters was developed and applied. There are many advantages of using multiple UAVs over a single UAV, so research and development for application to various fields are active. Ju and Son demonstrated that multiple UAVs are more efficient than single UAVs when using remote sensing in agriculture. It can reduce the overall flight time, simultaneously reducing battery consumption and increasing the required coverage area [20]. Gi et al. proposed a method for effective collaborative operation by combining a wireless sensor network and multiple UAVs and a method for recognizing objects and locations using multiple UAVs [23]. Paula et al. proposed a new modular solution for the autonomous driving of multiple UAVs. The platform can abstract control details completely, allowing inexperienced users to plan, execute, and monitor complex missions with one or more UAVs. Furthermore, devices such as parachutes and sirens could be added to UAVs and activated when abnormal behavior is detected [41]. Yao et al. used an optimal mission assignment method and multiple quadcopters to minimize the time required for spraying [42]. However, most of the studies using a large number of UAVs have been developed to overcome the general disadvantages of a single UAV (e.g., short flight time and coverage), and studies to improve phenotyping are scarce. When acquiring images for field phenotyping with a single UAV, if the object being measured moves or changes due to a change in lighting or wind, a measurement error may occur during the 3D reconstruction process. If it is possible to acquire images taken from multiple perspectives almost simultaneously (within 20 ms), measurement errors during the reconstruction process can be reduced to solve this problem. This study was designed to enable cooperative driving while collecting crop images from various perspectives almost simultaneously using multiple UAVs. Accordingly, it is expected that field phenotyping can be improved, and the accuracy decrease caused by external disturbances (e.g., light and wind) can be overcome. Taking images from multiple perspectives while driving multiple UAVs simultaneously requires linking the image collection device with multiple UAVs. It is necessary to develop devices and programs to allow multiple UAVs to simultaneously acquire images from multiple perspectives by operating the cameras’ image-capturing triggers when all UAVs arrive at the waypoint in the same row. Furthermore, additional verification experiments are required to determine whether multi-perspective image acquisition using multiple UAVs helps to improve field phenotyping accuracy by comparing the field phenotyping results for the same field based on a single image taken from a single UAV and a multi-perspective image taken from multiple UAVs. Additionally, the collision avoidance algorithm worked correctly, but when MAVSDK was applied to the actual aircraft, the return command did not work correctly, so it was replaced with a spot landing. The next plan is to solve this by replacing the FC and changing the firmware to Ardupilot. Finally, additional research is needed to measure the maximum distance of wireless communications for collaborative driving in a wider field, and to take countermeasures when wireless communications are disabled.

## 5. Conclusions

To minimize phenotypic errors by allowing multiple UAVs to simultaneously acquire images from different viewpoints and improve digital surface models, this study developed a novel system for the cooperation of multiple UAVs to optimize the cost, efficiency, and productivity, while reducing the impact on the external environment. The GCS developed in this system communicated wirelessly with different UAVs using MAVLink, and the loss rate and communication latency were 0% and 334 ms, respectively, which were similar to those of the common wireless communication method of UAVs. A GAZEBO-based simulation environment was developed to optimize the flight parameters of the collaborative driving algorithm and conduct the preliminary verification experiment prior to the field test. Through the simulation, the optimal parameter values (LBO: 0.4 m, flight speed: 3.0 m/s) were calculated. In field experiments conducted based on these parameters, it was found that two UAVs performed autonomous flight stably. The flight time and flight accuracy of the developed GCS were 185 s and 0.08 m, respectively, which increased the flight time by 58 s compared to the previous program (QgroundControl), but the flight accuracy was improved by 60%. The collaborative driving system developed in this study facilitates the collaborative driving of multiple UAVs, which cannot be performed by the existing commercial programs. Furthermore, because different UAVs are synchronized and fly autonomously by updating waypoints simultaneously, it is possible to acquire the images required for field phenotyping from multiple perspectives simultaneously, which can reduce errors in field phenotyping.

## Figures and Tables

**Table 1:** Input variables required for path generation.

Input Variables	Values
Site area	641 m^2^
Flight altitude	20 m
Overlap (side, front)	75%
Distance between UAV	3 m
Flight speed	4.0 m/s
Image size	3280 × 2464 pixels
Camera focal length	3.04 mm
Camera sensor size	4.6 mm

**Table 2:** Field experiment conditions and variables.

Description	Contents
Wind direction	South-west
Wind speed	1.0 m/s
Atmospheric temperature	30.0 °C
LBO	0.4 m
Maximum flight speed	3.0 m/s

**Table 3:** Field experiment conditions and variables.

Description	Contents
Wind direction	North-east
Wind speed	3.7 m/s
Atmospheric temperature	28.6 °C
LBO	0.4 m
Collision recognition distance	2.5 m
Maximum flight speed	3.0 m/s

**Table 4:** Simulation and real field comparison experimental variables.

Description	Values
Flight altitude	20 m
Overlap (side, front)	75%
Distance between UAVs	3 m
LBO	0.4 m
Flight speed	4.0 m/s
Image size	3280 × 2464 pixels
Camera focal length	3.04 mm
Camera sensor size	4.6 mm

**Table 5:** Flight performance comparison of simulation and actual field results.

	Simulation	Actual Field
Flight time	164 s	185 s
Flight accuracy	0.06 m	0.08 m

**Table 6:** Comparison of simulation and actual field results.

	QgroundControl	Developed Program
Flight time	127 s	185 s
Flight accuracy	0.2 m	0.08 m

## Data Availability

Please contact the corresponding author for data requests.

## References

[1] Maes W.H., Steppe K. (2019). Perspectives for remote sensing with unmanned aerial vehicles in precision agriculture. Trends Plant Sci..

[2] Tsouros D.C., Bibi S., Sarigiannidis P.G. (2019). A review on UAV-based applications for precision agriculture. Information.

[3] Fuentes-Peailillo F., Ortega-Farias S., Rivera M., Bardeen M., Moreno M. Comparison of vegetation indices acquired from RGB and multispectral sensors placed on UAV. Proceedings of the 2018 IEEE International Conference on Automation/XXIII Congress of the Chilean Association of Automatic Control (ICA-ACCA).

[4] Zheng H., Zhou X., He J., Yao X., Cheng T., Zhu Y., Cao W., Tian Y. (2020). Early season detection of rice plants using RGB, NIR-G-B and multispectral images from unmanned aerial vehicle (UAV). Comput. Electron. Agric..

[5] Kerkech M., Hafiane A., Canals R. (2018). Deep leaning approach with colorimetric spaces and vegetation indices for vine diseases detection in UAV images. Comput. Electron. Agric..

[6] Navia J., Mondragon I., Patino D., Colorado J. Multispectral mapping in agriculture: Terrain mosaic using an autonomous quadcopter UAV. Proceedings of the 2016 International Conference on Unmanned Aircraft Systems (ICUAS 2016).

[7] Christiansen M.P., Laursen M.S., Jørgensen R.N., Skovsen S., Gislum R. (2017). Designing and testing a UAV mapping system for agricultural field surveying. Sensors.

[8] Han X., Thomasson J.A., Swaminathan V., Wang T., Raman R., Rajan N., Neely H. (2020). Field-based calibration of unmanned aerial vehicle thermal infrared imagery with temperature-controlled references. Sensors.

[9] Han X., Thomasson J.A., Wang T., Swaminathan V. (2020). Autonomous mobile ground control point improves accuracy of agricultural remote sensing through collaboration with UAV. Inventions.

[10] López-Granados F., Torres-Sánchez J., De Castro A.I., Serrano-Pérez A., Mesas-Carrascosa F.J., Peña J.M. (2016). Object-based early monitoring of a grass weed in a grass crop using high resolution UAV imagery. Agron. Sustain. Dev..

[11] Ballesteros R., Ortega J.F., Hernandez D., Moreno M.A. (2018). Onion biomass monitoring using UAV-based RGB imaging. Precis. Agric..

[12] Koc-San D., Selim S., Aslan N., San B.T. (2018). Automatic citrus tree extraction from UAV images and digital surface models using circular Hough transform. Comput. Electron. Agric..

[13] Straffelini E., Cucchiaro S., Tarolli P. (2021). Mapping potential surface ponding in agriculture using UAV-SfM. Earth Surf. Process. Landf..

[14] Dileep M.R., Navaneeth A.V., Ullagaddi S., Danti A. A Study and analysis on various types of agricultural drones and its applications. Proceedings of the International Conference on Research in Computational Intelligence and Communication Networks (ICRCICN 2020).

[15] Ebeid E., Skriver M., Jin J. A Survey on open-source flight control platforms of unmanned aerial vehicle. Proceedings of the 2017 Euromicro Conference on Digital System Design (DSD).

[16] Nguyen T.T., Slaughter D.C., Townsley B.T., Carriedo L., Maloof J.N., Sinha N. In-field plant phenotyping using multi-view reconstruction: An investigation in eggplant. Proceedings of the 13th International Conference on Precision Agriculture.

[17] Avellar G.S.C., Pereira G.A.S., Pimenta L.C.A., Iscold P. (2015). Multi-UAV routing for area coverage and remote sensing with minimum time. Sensors.

[18] Engebraten S., Glette K., Yakimenko O. Field-testing of high-level decentralized controllers for a multi-function drone swarm. Proceedings of the IEEE International Conference on Control and Automation (ICCA 2018).

[19] Zaidi A., Kazim M., Weng R., Wang D., Zhang X. (2021). Distributed Observer-Based Leader Following Consensus Tracking Protocol for a Swarm of Drones. J. Intell. Robot. Syst..

[20] Ju C., Son H. (2018). Multiple UAV Systems for Agricultural Applications: Control, Implementation, and Evaluation. Electronics.

[21] Barrientos A., Colorado J., Cerro J., Martinez A., Rossi C., Sanz D., Valente J. (2011). Aerial remote sensing in agriculture: A practical approach to area coverage and path planning for fleetsof mini aerial robots. J. Field Robot..

[22] Roberge V., Tarbouchi M., Labonte G. (2013). Comparison of parallel genetic algorithm and particle swarm optimization for real-time UAV path planning. IEEE Trans. Ind. Inform..

[23] Gu J., Su T., Wang Q., Du X., Guizani M. (2018). Multiple moving targets surveillance based on a cooperative network for multi-UAV. IEEE Commun. Mag..

[24] Lee B.H.Y., Morrison J.R., Sharma R. Multi-UAV control testbed for persistent UAV presence: ROS GPS waypoint tracking package and centralized task allocation capability. Proceedings of the International Conference on Unmanned Aircraft Systems (ICUAS 2017).

[25] Greenwood F. (2016). Drones on the Horizon: New Frontier in Agricultural Innovation. ICT Update, Issue 82. https://cgspace.cgiar.org/bitstream/handle/10568/89779/ICT082E_PDF.pdf.

[26] Young S.N., Kayacan E., Peschel J.M. (2019). Design and field evaluation of a ground robot for high-throughput phenotyping of energy sorghum. Precis. Agric..

[27] Madec S., Baret F., De Solan B., Thomas S., Dutartre D., Jezequel S., Hemmerlé M., Colombeau G., Comar A. (2017). High-throughput phenotyping of plant height: Comparing unmanned aerial vehicles and ground lidar estimates. Front. Plant Sci..

[28] Mueller-Sim T., Jenkins M., Abel J., Kantor G. The Robotanist: A ground-based agricultural robot for high-throughput crop phenotyping. Proceedings of the IEEE International Conference on Robotics and Automation (ICRA).

[29] Manish R., Lin Y.C., Ravi R., Hasheminasab S.M., Zhou T., Habib A. (2021). Development of a miniaturized mobile mapping system for in-row, under-canopy phenotyping. Remote Sens..

[30] Torres-Sánchez J., López-Granados F., Borra-Serrano I., Manuel Peña J. (2018). Assessing UAV-collected image overlap influence on computation time and digital surface model accuracy in olive orchards. Precis. Agric..

[31] Holman F.H., Riche A.B., Michalski A., Castle M., Wooster M.J., Hawkesford M.J. (2016). High throughput field phenotyping of wheat plant height and growth rate in field plot trials using UAV based remote sensing. Remote Sens..

[32] Ruiz J.J., Diaz-Mas L., Perez F., Viguria A. (2013). Evaluating the accuracy of dem generation algorithms from uav imagery. Int. Arch. Photogramm. Remote Sens. Spat. Inf. Sci..

[33] Willkomm M., Bolten A., Bareth G. Non-destructive monitoring of rice by hyperspectral in-field spectrometry and UAV-based remote sensing: Case study of field-grown rice in North Rhine-Westphalia, Germany. Proceedings of the International Archives of the Photogrammetry, Remote Sensing and Spatial Information Sciences (ISPRS Archives 2016).

[34] Zhu R., Sun K., Yan Z., Yan X., Yu J., Shi J., Hu Z., Jiang H., Xin D., Zhang Z. (2020). Analysing the phenotype development of soybean plants using low-cost 3D reconstruction. Sci. Rep..

[35] Sodhi P., Vijayarangan S., Wettergreen D. In-field segmentation and identification of plant structures using 3D imaging. Proceedings of the IEEE/RSJ International Conference on Intelligent Robots and Systems (IROS).

[36] He J.Q., Harrison R.J., Li B. (2017). A novel 3D imaging system for strawberry phenotyping. Plant Methods.

[37] Li J., Tang L. (2017). Developing a low-cost 3D plant morphological traits characterization system. Comput. Electron. Agric..

[38] Zermas D., Morellas V., Mulla D., Papanikolopoulos N. (2020). 3D model processing for high throughput phenotype extraction—The case of corn. Comput. Electron. Agric..

[39] Atoev S., Kwon K.R., Lee S.H., Moon K.S. Data analysis of the MAVLink communication protocol. Proceedings of the International Conference on Information Science and Communications Technologies (ICISCT).

[40] Ramirez-Atencia C., Camacho D. (2018). Extending QGroundControl for automated mission planning of Uavs. Sensors.

[41] Paula N., Areias B., Reis A.B., Sargento S. Multi-drone Control with Autonomous Mission Support. Proceedings of the IEEE International Conference on Pervasive Computing and Communications Workshops.

[42] Yao L., Jiang Y., Zhiyao Z., Shuaishuai Y., Quan Q. A pesticide spraying mission assignment performed by multi-quadcopters and its simulation platform establishment. Proceedings of the IEEE Chinese Guidance, Navigation and Control Conference.

